# Long stem revision versus short stem revision with plate osteosynthesis for Vancouver type B2 periprosthetic femoral fracture: a comparative study of eighty five cases

**DOI:** 10.1007/s00264-024-06181-w

**Published:** 2024-04-23

**Authors:** Jian-Jiun Chen, Shih-Hsin Hung, Jia-You Liou, Wen-Chieh Chang, Kuei-Hsiang Hsu, Yu-Pin Su, Fang-Yao Chiu, Ming-Fai Cheng

**Affiliations:** 1 Department of Orthopedics and Traumatology, Taipei Veterans General Hospital Shipai Rd Beitou Dist, No. 201, Sec. 2, Taipei City, 112201 Taiwan Republic of China; 2 Department of Orthopedic Surgery, School of Medicine, National Yang Ming Chiao Tung University, Taipei, Taiwan; 3 Department of Nursing, Taipei Veterans General Hospital, Taipei, Taiwan; 4 Department of Nursing, Chang Jung Christian University, Tainan, Taiwan; 5 Department of Medical Education, Taipei Veterans General Hospital, Taipei, Taiwan

**Keywords:** Periprosthetic femoral fractures, Hip, Vancouver B, Revision arthroplasty, Cementless long stem, Cementless short taper stem with plate osteosynthesis, Surgical techniques

## Abstract

**Purpose:**

Periprosthetic femoral fractures (PPFs) around the hip are challenging complications in orthopaedic surgery, particularly Vancouver type B2 (VTB2) fractures. The surgical management of these fractures is crucial and depends on various factors. Cementless short taper stem with plate osteosynthesis is an alternative surgical technique. This study aims to compare the outcomes of this surgical technique with revision arthroplasty (RA) with long stem in the treatment of VTB2 PPFs.

**Methods:**

This retrospective study was conducted in a single medical institute from February 2010 to May 2019. Patients who had received either total hip arthroplasty or bipolar hemiarthroplasty and subsequently developed a VTB2 PPF were included; patients who sustained intra-operative fractures or received a cemented stem previously were excluded from the analysis. The patients were divided into two groups: group I received RA with cementless long stem, while group II underwent RA with cementless short taper stem with plate osteosynthesis. Demographic data, radiographic and functional outcomes, and complications were analyzed between the two groups.

**Results:**

A total of 85 patients diagnosed with VTB2 PPFs were included in the study. There were no significant differences between the two groups in terms of demographic data, including age, gender, mean follow-up times, estimated blood loss, and operative times. The radiographic results showed that there was no significant difference in the incidence of subsidence and implant stability between the two groups. However, group II tended to have less subsidence and periprosthetic osteolysis. Patients in group II had significantly better functional scores (mean Harris hip score: post-operative: 60.2 in group I and 66.7 in group ii; last follow-up: 77.4 in group 1 and 83.2 in group II (both *p* < 0.05)). There were no significant differences in the overall complication rate, including infection, dislocation, re-fracture, and revision surgery, between the two groups.

**Conclusions:**

Both surgical techniques, cementless long stem and cementless short taper stem with plate osteosynthesis, are effective in the treatment of Vancouver B2 PPFs, with no significant differences in outcomes or complications. However, patients in cementless short taper stem with plate osteosynthesis had better functional scores at both post-operative and the last follow-up.

## Introduction

Periprosthetic femoral fractures (PPFs) are a serious complication that can arise after total hip arthroplasty (THA) or bipolar hemiarthroplasty procedures [4, 18]. These fractures occur around the prosthetic components in the hip joint and can lead to pain, instability, and loss of function [25]. As hip replacement surgeries become more prevalent, especially among the aging population, the incidence of periprosthetic fractures is expected to rise [8, 29].

The Vancouver classification system is widely employed for categorizing periprosthetic hip fractures, aiding in treatment decision-making based on the fracture's location and characteristics [7, 19, 22]. According to the Vancouver algorithm, open reduction and internal fixation (ORIF) utilizing a locking compression plate (LCP) represent a well-accepted and established treatment modality for Vancouver type B1 (VTB1) fractures [15, 20]. In cases involving a loose stem and compromised bone quality, such as VTB3 fractures, revision arthroplasty (RA) employing a long stem to bypass the fracture and augmentation remains the recommended surgical approach [14, 17].

However, Vancouver type B2 PPFs, which involve a fracture around or just below the stem with a loose stem but good proximal bone, present the most contentious classification in current treatment approaches. This is because VTB1 fractures typically undergo direct ORIF, while VTB3 fractures receive direct revision. In contrast, VTB2 falls in between, making it eligible for both ORIF and revision. The academic community has seen numerous studies discussing the differences between ORIF and revision when applied to VTB2 patients [2, 10, 28]. Nevertheless, there is room for improvement in the outcomes of both approaches.

Considering the complexity of the injury and the potential for significant bone loss, we have opted for a different combination of surgical techniques, including revision arthroplasty and internal fixation.

This retrospective study compares the outcomes of two surgical treatments for Vancouver type B2 PPFs: revision arthroplasty with cementless long stem (group I) and cementless short taper stem with plate osteosynthesis (group II). The aim of this study is to determine which of the two surgical techniques provides better outcomes for patients with Vancouver type B2 PPFs. We hypothesize that there will be no significant differences in outcomes or complications between the two groups. However, we expect that patients in group II will have better functional scores at both post-operative and the last follow-up. This study has the potential to inform surgical decision-making and improve patient outcomes for Vancouver type B2 PPFs.

## Materials and methods

### Methods

This retrospective analysis was conducted at a tertiary medical institute and was approved by the Institutional Review Board of Taipei Veterans General Hospital (2024–03-005BC). This study examined patient records from February 2010 to May 2019, aiming to investigate the outcomes of patients who had undergone total hip arthroplasty or bipolar hemiarthroplasty and subsequently experienced a secondary Vancouver type B2 PPFs.

Inclusion criteria for the study were patients who older than 50 years old had received either total hip arthroplasty or bipolar hemiarthroplasty and subsequently developed a Vancouver type B2 periprosthetic fracture. Exclusion criteria included malignant metastasis with hip arthroplasty, previous hip surgery with cemented stem, intraoperative periprosthetic fractures, patients who could not cooperate with follow-up, and follow-up less than two years. The reason for exclusion of patients with cemented stem was due to the relatively small number of patients with this type of stem at the institute.

**Fig. 1:**
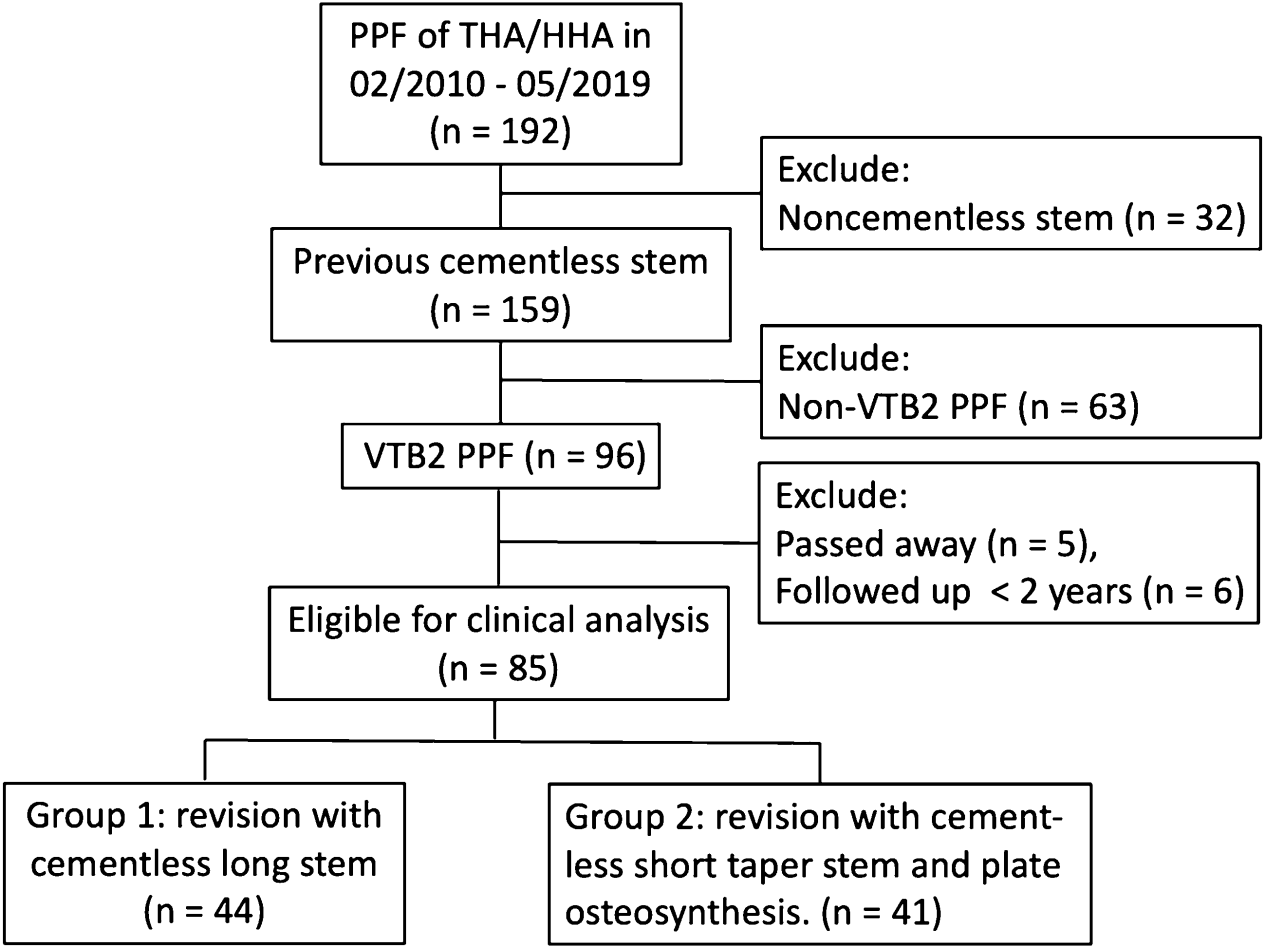
Clinical trial profile and patient flowchart. During the enrollment period (February 2010 to May 2019), a total of 192 patients underwent surgery for PPF following THA or HHA at our institution. Exclusions were made for 32 patients using noncementless stems, 63 patients with PPF not classified as VTB2, and 11 patients with less than 2 years of follow-up (five of whom were deceased). The final analysis included 85 eligible patients, who were further divided into two groups: the first group underwent revision with a cementless long stem (44 patients), while the second group underwent revision with a cementless short taper stem and plate osteosynthesis (41 patients)

During the study period, 192 patients were diagnosed with periprosthetic fractures after primary total hip arthroplasty or bipolar hemiarthroplasty. Among these patients, 159 had been treated with a cementless stem, and 96 were confirmed to have Vancouver type B2 PPFs. The study ultimately included 85 patients diagnosed with Vancouver B PPFs, as five patients passed away and six patients were followed up for less than two years, which disqualified them from the analysis. There was no missing data among these 85 patients (Fig. 1).

### Patient grouping

The study participants were divided into two groups based on the surgical approach chosen by two experienced orthopaedic surgeons specializing in hip arthroplasty and trauma. Forty-four patients were divided into group I which underwent RA using a cementless long stem (U2 revision stem, United®), while 41 patients were divided into group II which received RA with a cementless short taper stem (Zimmer® M/L Taper Hip Prosthesis) and rigid fixation with plate osteosynthesis (DePuy Synthes, Variable Angle Condylar LCP). Both groups underwent open reduction with cerclage wiring to reduce fractures and remove the previous stem.

### Surgical technique

#### **Group I (Long Stem only) (**Fig. 2**)**

**Fig. 2:**
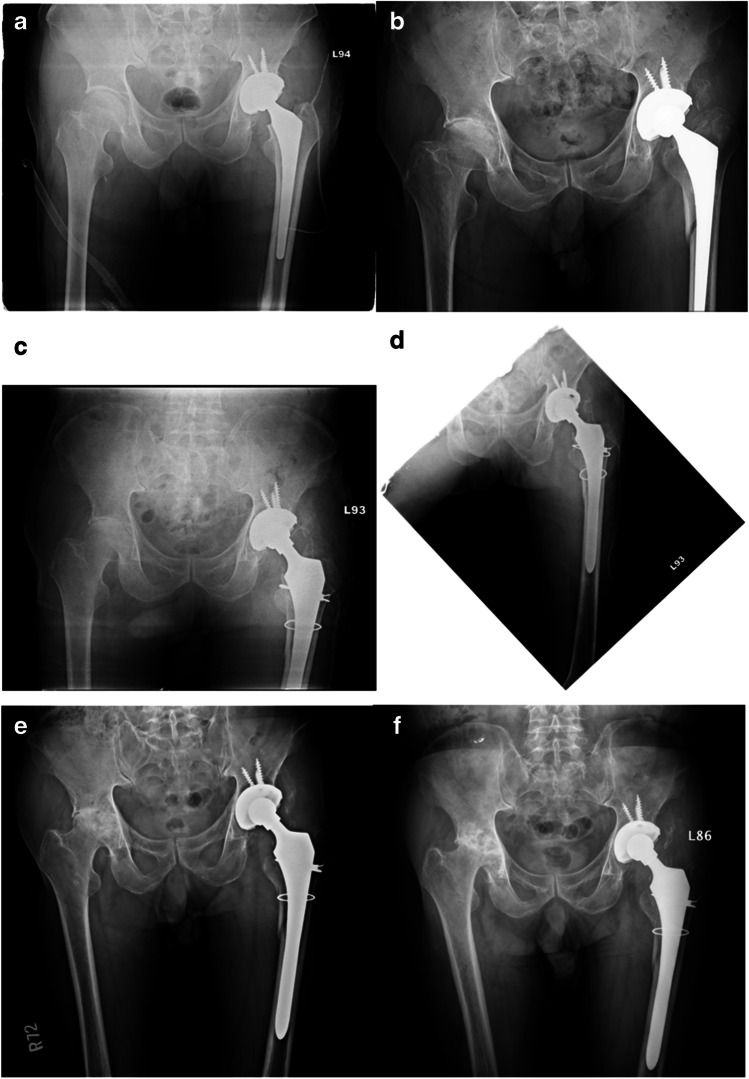
The series of events and treatments for a 78-year-old female patient who underwent a left total hip arthroplasty and subsequently experienced complications: **a** Initial: The patient initially received a left total hip arthroplasty. **b** Complication: The patient suffered an accidental fall, leading to a post left total hip arthroplasty complication—a peri-prosthetic fracture, classified as VTB2. **c**, **d** Revision surgery: To address the complication, the patient underwent a revision surgery using a cementless long stem to replace the loosening prosthesis. **e** 12-month follow-up: After 12 months of recovery, the patient experienced shortening of the affected limb with a subsidence of 8 mm. However, some bridging callus formation was observed around the fracture site, indicating healing progress. **f** 15-month follow-up: at the 15-month follow-up, the patient’s stem had not subsided further and was in a stable condition. He was able to return to her daily activities

In the surgical approach of group 1, we initiate the procedure by creating a straight lateral thigh incision through the posterolateral approach, involving the incision of the tensor fascia lata and the separation of the gluteus maximus to gain access to the hip joint. Following this, the femoral head is dislocated, and the vastus lateralis muscle is elevated and retracted to expose the fracture site. Subsequently, the loosening stem is removed, and cerclage wiring is employed for fracture reduction, ensuring the placement of at least two sets of wires above and below the lesser trochanter. The femoral canal is then meticulously prepared using a flexible reamer, tailored to the appropriate size based on the canal dimensions. A trial of the long stem is conducted through broaching, and the fit is carefully assessed using intraoperative fluoroscopy. Finally, the hip joint is reduced, and a comprehensive evaluation is performed to gauge its stability and assess the tension of the surrounding soft tissues.

#### **Group II (short taper stem** + **ORIF with LCP) (**Fig. 3**)**

**Fig. 3:**
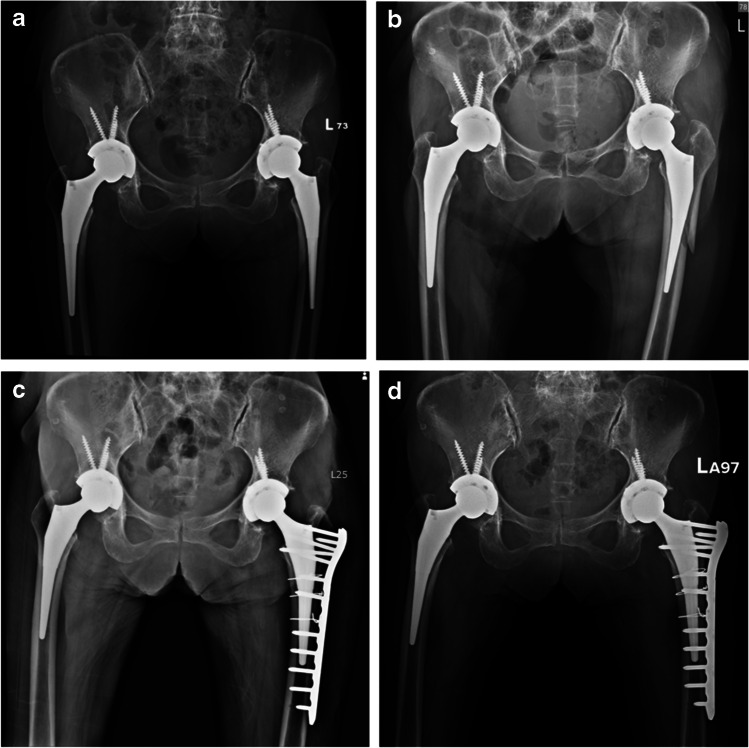
The series of events and treatments for an 83-year-old female patient who underwent a left total hip arthroplasty and subsequently experienced complications: **a** Initial: The initial x-ray image shows the patient's hip joint after receiving a left total hip arthroplasty. **b** Complication: The patient suffered an accidental fall, which resulted in a post left total hip arthroplasty complication—a peri-prosthetic fracture, classified as VTB2. A fracture around the stem of the implant with a loose prosthesis, requiring revision surgery. **c** Revision surgery: To address the complication, the patient underwent a revision surgery. A short taper stem was used to replace the damaged prosthesis, and open reduction internal fixation with a VA condylar locking compression plate was performed to stabilize the fracture. **d** Follow-up: After 12 months of follow-up, the patient showed no signs of stem subsidence or implant failure. She was able to return to her daily activities without any significant issues

In the surgical approach of group 2, a straight lateral thigh incision is made through the posterolateral approach, involving the incision of the tensor fascia lata and the separation of the gluteus maximus to gain access to the hip joint. Subsequently, the femoral head is dislocated, and the vastus lateralis muscle is elevated and retracted to expose the fracture site. The loosening stem is then removed, and cerclage wiring is utilized for fracture reduction, ensuring the placement of at least two sets of wires above and below the lesser trochanter. To achieve a press fit, the stem is revised to a larger size using a short taper stem trial, such as the Zimmer M-L taper stem. Additionally, a contra-lateral distal femur VA condylar LCP is positioned laterally to the greater trochanter tip along the femur shaft and secured with four locking screws. Finally, the hip joint is reduced, and a comprehensive assessment is conducted to evaluate its stability and the tension of the surrounding soft tissues.

### Post-operative protocol

Group I patients were required to ambulate with protective weight bearing and crutches for six weeks, followed by partial weight bearing for another six weeks before being allowed full weight bearing. Group II patients were allowed immediate full weight bearing.

### Outcome measures

The outcome measures for this study included the radiographic and clinical outcomes of the patients. Radiographic outcomes were assessed at last follow-up, including assessments of stem migration, periprosthetic osteolysis, and implant stability. Clinical outcomes were assessed post-operatively and at last follow-up, using the visual analog scale (VAS) for pain and Harris hip scores (HHSs). VAS scores were assessed to measure pain levels on a scale from 0 to 10. HHSs were used with scores ranging from 0 to 100, where higher scores indicate better functional outcomes and lower scores indicate poorer functional outcomes [31].

Statistical analysis descriptive statistics were used to summarize the demographic data of the patients. The clinical and radiographic outcomes of the two groups were compared using independent sample *t* tests and chi-square tests. A *p* value of less than 0.05 was considered statistically significant.

## Results

### Demographic data and clinical characteristics

Table 1 summarizes the demographic and clinical characteristics of the patients in group I (revision with long stem) and group II (revision with short taper stem + ORIF). There were no significant differences between the two groups in terms of age, gender, injury mechanism, follow-up time, fracture classification, operative time, intra-operative blood loss, transfusion units, hospital stay, and return to walk.

**Table 1:** Demographic and clinical characteristics of the patients

Variables	Group I (*n* = 44)	Group II (*n* = 41)	*P* value
Age (years)	74.2 ± 7.6	73.9 ± 8.1	0.836
Gender (male/female)	18/26	19/22	0.721
Injury mechanism (fall/trauma)	24/20	21/20	0.805
Follow-up time (mo)	71.3 ± 32.2 (24–155)	69.0 ± 31.2 (29–128)	0.637
Fracture classification (B2.1/B2.2/B2.3)	11/22/11	10/23/8	0.845
Operative time (min)	148.9 ± 36.7	144.2 ± 32.4	0.491
Intra-operative blood loss (ml)	326.4 ± 127.1	320.3 ± 121.6	0.756
Transfusion units (*n*)	2.3 ± 1.5	2.2 ± 1.6	0.804
Hospital stay (days)	15.2 ± 4.6	14.7 ± 4.3	0.595
Return to walk (days)	8.6 ± 2.1	8.1 ± 2.0	0.188

Statistical analysis of the radiographic parameters of these 2 groups was performed using the Mann–Whitney rank sum test, and the unpaired *t* test from the SigmaStat software package, version 2.0 (Jandel Corp., San Rafael, CA), and power analysis was also performed with the alpha set at 0.05. A *p* value less than 0.05 was considered significant

### Radiological outcomes

Radiographic evaluation was performed to compare the subsidence, radiolucent line in Gruen zone (analyzed in AP view), periprosthetic osteolysis, and implant stability between the two groups. Table 2 shows the radiographic outcomes of the patients in group I and group II. The results showed that the mean subsidence in group I was 2.1 ± 0.8 mm, while it was 1.9 ± 0.6 mm in group II. Although there was no significant difference between the two groups, group II tended to have less subsidence. Regarding the radiolucent line in Gruen zone, group I had six cases, while group II had three cases, but the difference was not significant. The incidence of periprosthetic osteolysis was 8 cases in group I and four cases in group II, which showed a trend that group II had fewer cases, but the difference was not significant. The implant stability was 42 cases in group I and 39 cases in group II, and there was no significant difference between the two groups.

**Table 2:** Radiographic outcomes and clinical outcomes of the patients

	Group I (*n* = 44)	Group II (*n* = 41)	*P* value
Radiographic outcomes Subsidence (mm)	2.1 ± 0.8	1.9 ± 0.6	0.122
Radiolucent line in Gruen zone (*n*)	6	3	0.181
Periprosthetic osteolysis (*n*)	8	4	0.082
Implant stability (*n*)	42	39	0.467
Clinical outcomes VAS score (post-operative)	5.1 ± 1.2	5.2 ± 1.0	0.680
VAS score (last follow-up)	3.8 ± 0.9	3.6 ± 0.8	0.096
Harris hip score (post-op)	60.2 ± 7.5	66.7 ± 9.3	0.001
Harris hip score (last f/u)	77.4 ± 8.3	83.2 ± 6.5	< 0.001
Complications			
Overall complications (*n*)	7	5	0.512
Infection (*n*)	2	1	0.601
Dislocation (*n*)	1	0	0.495
Re-fracture (*n*)	2	2	0.999
Revision surgery (*n*)	2	2	0.999

Statistical analysis of the radiographic parameters of these 2 groups was performed using the Mann–Whitney rank sum test, and the unpaired *t* test from the SigmaStat software package, version 2.0 (Jandel Corp., San Rafael, CA), and power analysis was also performed with the alpha set at 0.05. A *p* value less than 0.05 was considered significant

### Clinical outcomes

Clinical outcomes of the patients in group I and group II are shown in Table 2. There were no significant differences in the visual analog scale (VAS) scores at the last follow-up between the two groups. However, patients in group II had better functional outcomes at both post-operative and the last follow-up. The mean Harris hip score (HHS) was 60.2 ± 7.5 in group I and 66.7 ± 9.3 in group II at post-operative (*p* = 0.001); 77.4 ± 8.3 in group 1 and 83.2 ± 6.5 in group II at the last follow-up (*p* < 0.001).

### Complications

Table 2 shows the complications of the patients in group I and group II. There were no significant differences in the overall complication rate, including infection, dislocation, re-fracture, and revision surgery, between the two groups.

## Discussion

In summary, the study included a total of 85 patients diagnosed with VTB2 PPFs, each with a minimum follow-up period of two years. Radiographic analysis revealed the trend of less subsidence and periprosthetic osteolysis in group II, which might suggest that cementless short taper stem with plate osteosynthesis might provide better initial stability and implant-bone interface than cementless long stem. Patients in group II exhibited significantly better functional scores (HHSs) compared to group I at both post-operative and the last follow-up, which may be attributed to the additional stability provided by plate osteosynthesis, allowing for more rapid rehabilitation and weight-bearing post-operatively.

The treatment of Vancouver type B2 PPFs is a challenging task for orthopaedic surgeons. The surgical management of these fractures depends on many factors, such as the patient’s age, medical condition, fracture classification, and the experience of the surgeon. The current mainstream approaches can be divided into two categories: the first is ORIF, which involves reinforcing fixation around the PPFs using steel plates. The advantages of ORIF include being a less invasive surgical procedure with reduced surgery time and blood loss, along with lower implant costs. Additionally, it allows for the possibility of future stem revision [21, 27]. However, concerns about the stability of fixation may arise with ORIF, especially when considering that the stem and bone interface is already compromised. ORIF does not directly address this issue. Moreover, radiographic outcomes following ORIF have been suboptimal based on Beals and Tower’s criteria [27] and associated with higher rate of subsidence [10]. The second approach is revision arthroplasty, where a new artificial joint is created. The alternative artificial joint typically incorporates a long stem for stability [2, 10, 14, 28]. This method which bypasses the fracture offers the advantage of directly addressing the instability between the stem and bone while securing the fracture site, remains the recommended surgical procedure for the challenging Vancouver type B2 PPFs [1, 6, 24, 30]. However, it has the drawback of causing more significant damage to the femoral bone stock, making future RA procedures more challenging [11, 13]. ORIF was performed for certain type injuries in patients who were not medically fit for RA, particularly when dealing with elderly patients with multiple comorbidities, while RA was chosen for other cases [5, 9, 12, 26, 33]. Previous meta-analysis including 1621 patients demonstrates that RA has a similar revision and complication rate to ORIF in Vancouver B2 PFFs [16].

In our study, we employed an alternative surgical approach beyond the two previously mentioned methods: the combination of RA along with ORIF. Specifically, we utilized a cementless short taper stem with plate osteosynthesis for the treatment of Vancouver type B2 PPFs. This approach is aimed at harnessing the advantages of both the preceding methods: preserving more bone stock through the use of a short stem while enhancing the fixation effect achieved by ORIF. It is noteworthy that such a surgical technique is currently not documented in existing literature. In this study, we compared the outcomes of RA with cementless long stem (group I) and cementless short taper stem with plate osteosynthesis (group II) in the treatment of Vancouver type B2 PPFs. Our results showed that both surgical techniques are effective, with no significant differences in outcomes or complications between the two groups.

In terms of radiological outcomes, current literature suggests that the utilization of ORIF may yield results on par with those achieved through stem revision [23, 28]. When addressing VTB2 PPF, Solomon et al. demonstrated complete fracture healing and stable stem conditions in the ORIF group based on radiographic assessments, with no radiological complications documented in the revision cohort [26]. In a review study conducted by Haider et al., a lower rate of subsidence was observed in the RA group compared to ORIF for VTB2 PPF, with no significant difference was noted in terms of loosening [10]. Our results further indicating that group II tended to have less subsidence and periprosthetic osteolysis, which suggests that cementless short taper stem with plate osteosynthesis might provide better initial stability and implant-bone interface than cementless long stem.

Regarding functional outcomes, Haider et al. demonstrated no significant differences in clinical outcome and the percentage of patients achieving full weight-bearing when comparing RA and ORIF for the treatment of Vancouver type-B2 fractures [10]. In our study, the better functional outcomes observed in group II at both post-operative and the last follow-up may be attributed to the additional stability provided by plate osteosynthesis, which could allow for more rapid rehabilitation and weight-bearing post-operatively. Furthermore, the implementation of a short taper stem could lead to reduced disruption of the femoral bone stock while simultaneously decreasing thigh pain and proximal stress shielding [3, 32]. These factors may potentially contribute to enhance both short-term and long-term functional outcomes.

Currently, the surgical management of challenging VTB2 periprosthetic fractures primarily involves RA with a long stem, while ORIF may be considered for patients with multiple comorbidities. Our proposed alternative technique has shown outcomes slightly superior to those of revision with a long stem. Although we did not specifically compare the outcomes of short stem + ORIF with those of sole ORIF, existing literature indicates that the efficacy of revision with a long stem and ORIF is comparable [2, 10, 16, 28]. In summary, we hypothesize that the effectiveness of short stem + ORIF is not inferior to that of sole ORIF or sole revision with a long stem. This could be considered as one of the options for future patient care.

## Limitation

There are several limitations to our study. Firstly, the retrospective design of the study and the relatively small sample size might have resulted in selection bias and insufficient statistical power to detect significant differences between the two groups. Secondly, the follow-up period of at least 2 years might not be long enough to capture potential differences in the long-term survivorship of the implants. Finally, there might be unmeasured confounding variables that could have influenced the outcomes and complications.

## Conclusion

In conclusion, our study compared the outcomes of revision arthroplasty with cementless long stem and cementless short taper stem with plate osteosynthesis in the treatment of Vancouver type B2 PPFs. Both surgical techniques are effective, with no significant differences in outcomes or complications between the two groups. However, patients underwent cementless short taper stem with plate osteosynthesis, had better functional outcomes at both post-operative and the last follow-up. These findings might help inform surgical decision-making and improve patient outcomes for Vancouver type B2 PPFs. Further research including prospective studies with larger sample sizes and longer follow-up periods is needed.
